# The urgent need for arbovirus surveillance and control following a catastrophic event: The case of the DANA flood event in Valencia

**DOI:** 10.1016/j.nmni.2024.101547

**Published:** 2024-11-29

**Authors:** Giancarlo Ceccarelli, Francesco Branda, Marta Giovanetti, Massimo Ciccozzi, Fabio Scarpa

**Affiliations:** Department of Public Health and Infectious Diseases, University Hospital Policlinico Umberto I, Sapienza University of Rome, Rome, Italy; Migrant and Global Health Research Organization (Mi-HeRO), Rome, Italy; Unit of Medical Statistics and Molecular Epidemiology, Università Campus Bio-Medico di Roma, Rome, Italy; Sciences and Technologies for Sustainable Development and One Health, Università Campus Bio-Medico di Roma, Rome, Italy; Instituto Rene Rachou, Fundação Oswaldo Cruz, Minas Gerais, Brazil; Unit of Medical Statistics and Molecular Epidemiology, Università Campus Bio-Medico di Roma, Rome, Italy; Department of Biomedical Sciences, University of Sassari, Sassari, Italy

Dear Editor,

The heavy rainfall and extensive flooding caused by the DANA (Depresión Aislada en Niveles Altos) [1] event in the Valencian Community have created numerous breeding sites for *Aedes albopictus*, a highly efficient vector for arboviruses such as dengue, Zika, and chikungunya. Since its establishment in Valencia in 2015, *Ae. albopictus* has posed an increasing public health threat, with its population and geographical distribution expanding significantly over the years. The predicted temperatures of 16–22 °C in the days following the floods further exacerbate the risk, as this range is optimal for mosquito reproduction and arbovirus transmission [2]. Combined with the vast amounts of stagnant water left by the floods, which serve as ideal larval habitats, this situation represents a pressing public health challenge for Valencia (Fig. 1) [3].

**Fig. 1:**
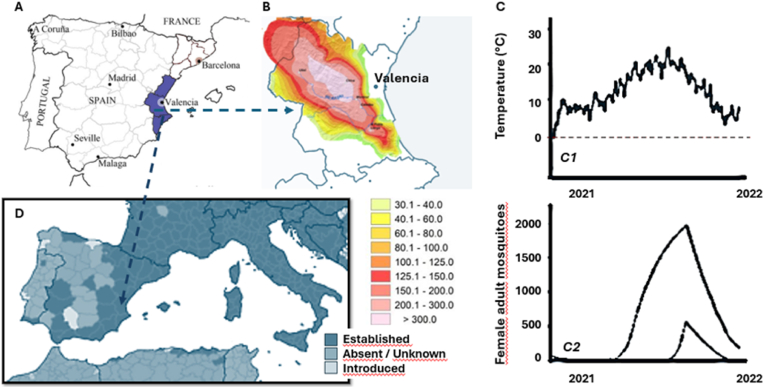
A) Map of Spain and the Valencian Community; B); Accumulated precipitation on October 29, 2024 (l/m^2^) reported by the Agencia Estatal de Meteorología (Source: World Meteorological Organization (WMO). Devastating rainfall hits Spain in yet another flood-related disaster. (2024) Available from https://wmo.int/media/news/devastating-rainfall-hits-spain-yet-another-flood-related-disaster accessed on 4/11/2024). C) Modeled vector population as a function of temperature in Spain, 2021: (C1) displays the average temperature across the 26 Spanish provinces included in the study, (C2) shows the modeled adult female vector population for these provinces. The black lines represent the highest and lowest observed vector populations; D) Current known distribution of invasive *Aedes* mosquito species (*Ae. aegypti, Ae. albopictus, Ae. atropalpus, Ae. japonicus,* and *Ae. koreicus*) in Spain at the regional administrative level, as of July 2024. (Source: European Centre for Disease Prevention and Control and European Food Safety Authority. Mosquito maps [internet]. Stockholm: ECDC; 2024. Available from: https://ecdc.europa.eu/en/disease-vectors/surveillance-and-disease-data/mosquito-maps accessed on 4/11/2024).

The DANA floods represent a textbook example of how climate extremes can interact with vector ecology to increase the risk of arboviral outbreaks. Beyond the immediate temperature and water-related factors, the aftermath of floods often leads to disruptions in waste management systems and sanitation, creating additional mosquito breeding opportunities in urban and peri-urban areas. Studies have shown that urbanized environments with limited drainage infrastructure, such as many neighborhoods in Valencia, can significantly enhance *Ae. albopictus* proliferation [3]. Furthermore, the impact of climate change on weather patterns, including more frequent and severe flooding events, has created a favorable environment for the establishment and spread of invasive mosquito species like *Ae. albopictus* across Southern Europe. In this context, Valencia serves as a sentinel for understanding and mitigating these broader environmental health risks.

Between 2016 and 2018, Valencia recorded 21 imported arbovirus cases (8 dengue, 7 chikungunya, and 6 Zika), each of which required intensive entomological surveillance and control measures to limit the risk of local transmission. In 38 % of these cases, *Ae. albopictus* was detected in the surrounding risk zones, highlighting its potential to serve as a bridge vector between imported cases and local outbreaks [4,5]. These data not only demonstrate the capacity for imported cases to trigger localized transmission but also emphasize the importance of preemptive action to manage the vector population, particularly in the wake of environmental crises like the DANA floods.

To minimize the risk of arbovirus outbreaks following catastrophic events such as DANA, a multifaceted strategy combining immediate interventions and long-term measures is needed, as summarized in Table 1.

**Table 1:** Intervention strategies to mitigate the risks of arbovirus outbreaks following catastrophic events such as the DANA floods.

Intervention strategy	Description	Key actions
Enhanced mosquito surveillance	Strengthen monitoring efforts to identify high-risk areas and track mosquito populations after flooding events.	- Map stagnant water sources such as natural pools, clogged drainage systems, and abandoned containers. - Deploy advanced tools like real-time PCR to detect arboviruses in mosquitoes. - Monitor environmental parameters like temperature and humidity to predict breeding cycles.
Scalable vector control interventions	Reduce mosquito populations through a combination of chemical and biological control measures.	- Use larvicides to target mosquito larvae and prevent maturation into adults. - Apply environmentally safe agents like *Bacillus thuringiensis israelensis* (Bti) in sensitive ecosystems. - Conduct adulticiding using ultra-low volume (ULV) sprays in urban areas with high mosquito activity. - Establish regular vector control schedules to maintain low mosquito density over time.
Public health campaigns and community engagement	Educate and empower local communities to reduce breeding sites and protect themselves from mosquito bites.	- Launch awareness campaigns using social media, local radio, and schools to inform residents about preventive measures. - Distribute educational materials on eliminating standing water, proper waste disposal, and use of mosquito repellents. - Partner with community leaders and organizations to promote active participation and create neighborhood-based mosquito control initiatives.
Preparedness for arbovirus cases	Develop a comprehensive system for rapid detection, response, and management of arbovirus infections to prevent outbreaks.	- Ensure widespread availability of diagnostic tests for arboviral diseases at healthcare facilities. - Train healthcare workers to identify symptoms and provide early treatment for dengue, Zika, and Chikungunya. - Implement immediate vector control in areas surrounding confirmed cases to halt local transmission. - Maintain an updated database of cases to facilitate real-time risk mapping and resource allocation.

One of the key lessons learned from the DANA event is the need for long-term investment in urban planning and infrastructure. Effective flood management requires improved drainage systems, stormwater control, and sound waste management infrastructure. These measures are essential to reduce the accumulation of standing water, the main breeding ground for mosquitoes. However, infrastructure alone is not enough. It must be accompanied by innovations in public health planning that are informed by real-time climate data, predictive modeling, and monitoring of vector populations. Using advanced technologies, authorities can anticipate possible outbreaks and reduce risks long before they escalate. This shift from a reactive to a proactive approach can dramatically reduce the likelihood of large-scale disease transmission.

Moreover, addressing arboviral threats requires a multifaceted approach that includes environmental management, robust surveillance, and health education. In Valencia, awareness campaigns could be designed to raise awareness about the dangers of standing water and the importance of individual protective measures such as insect repellents and mosquito nets. Community involvement is critical in this regard, as empowering local residents to take charge of vector control measures helps create a collective defense against the disease. When communities are informed and involved, they can take simple but effective actions, such as eliminating standing water in their homes, significantly reducing breeding sites for *Ae. albopictus*.

The economic implications of inaction in the face of potential arbovirus outbreaks are profound. In addition to health impacts, the social and economic costs of an outbreak could be devastating. An outbreak of dengue, Zika, or chikungunya could cause major disruptions to daily life, overburden local health systems, and discourage tourists, negatively impacting the economy, particularly in a region like Valencia, known for its vibrant tourism industry. Moreover, localized outbreaks could also affect key sectors such as agriculture and trade, leading to long-term economic challenges. Therefore, preemptive and coordinated public health measures are not only necessary to protect human health, but also to preserve the region's economic well-being.

Valencia's experience during the DANA event offers a microcosmic view of the broader challenges faced by regions in southern Europe and other parts of the world as climate change intensifies extreme events. Rising temperatures, erratic precipitation, and changing weather patterns are set to exacerbate the frequency and severity of extreme weather events, creating more favorable conditions for the spread of invasive mosquito species. This makes it even more imperative that public health authorities adapt and build resilience against such challenges. Integrating climate and environmental health data into public health planning can help predict potential risks and enable more strategic responses.

In conclusion, the DANA flood event in Valencia underscores the urgency of a holistic, forward-looking approach to public health in the face of climate change. It represents an opportunity to implement evidence-based measures to prevent arbovirus outbreaks while improving resilience to future climate change risks. By prioritizing surveillance, vector control, community involvement, and long-term infrastructure investments, Valencia can serve as a model for other flood-vulnerable regions seeking to mitigate the health impacts of extreme weather events. Lessons learned from this event should serve as a wake-up call for global public health systems to adopt proactive strategies that anticipate and address the interconnected challenges of climate change, urbanization, and vector-borne diseases.

## CRediT authorship contribution statement

**Giancarlo Ceccarelli:** Conceptualization, Investigation, Visualization, Writing – original draft, Writing – review & editing. **Francesco Branda:** Investigation, Writing – original draft, Writing – review & editing. **Marta Giovanetti:** Writing – original draft, Writing – review & editing. **Massimo Ciccozzi:** Supervision, Validation, Writing – original draft, Writing – review & editing. **Fabio Scarpa:** Writing – original draft, Writing – review & editing.

## Declaration of competing interest

The authors declare that they have no known competing financial interests or personal relationships that could have appeared to influence the work reported in this paper.
